# Light and temperature shape nuclear architecture and gene expression

**DOI:** 10.1016/j.pbi.2018.05.018

**Published:** 2018-10

**Authors:** Eirini Kaiserli, Giorgio Perrella, Mhairi LH Davidson

**Affiliations:** Institute of Molecular, Cell and Systems Biology, College of Medical, Veterinary and Life Sciences, Bower Building, University of Glasgow, Glasgow G12 8QQ, UK

## Abstract

Environmental stimuli play a major role in modulating growth and development throughout the life-cycle of a plant. Quantitative and qualitative variations in light and temperature trigger changes in gene expression that ultimately shape plant morphology for adaptation and survival. Although the phenotypic and transcriptomic basis of plant responses to the constantly changing environment have been examined for decades, the relationship between global changes in nuclear architecture and adaption to environmental stimuli is just being uncovered. This review presents recent discoveries investigating how changes in light and temperature trigger changes in chromatin structure and nuclear organization with a focus on the role of gene repositioning and chromatin accessibility in regulating gene expression.

## Highlights

• Arabidopsis and rice genomes exhibit diversity in chromatin organization in three-dimension.
• Light triggers changes in genome topology and transcriptional activity.
• Plant photoreceptors control nuclear morphology and chromatin architecture.
• Temperature fluctuations modify local chromatin structure and transcriptional activity.

**Current Opinion in Plant Biology** 2018, **45**:103–111

This review comes from a themed issue on **Cell signalling and gene regulation**

Edited by **Jorge Casal** and **Javier Palatnik**

For a complete overview see the Issue and the Editorial

Available online 15th June 2018

**https://doi.org/10.1016/j.pbi.2018.05.018**

1369-5266/© 2018 The Authors. Published by Elsevier Ltd. This is an open access article under the CC BY license (http://creativecommons.org/licenses/by/4.0/).

## Introduction

Plants perceive and utilize information from diverse environmental stimuli ranging from light quality, intensity, direction, photoperiod, temperature gradients, nutrient availability as well as biotic factors to optimize vegetative and reproductive success. The role of signal integration at the sub-cellular level is important for coordinating the biological processes that shape plant morphology, growth and survival in response to favourable but also adverse environmental conditions. Environmental stimuli trigger developmental and metabolic re-programming that primarily stem from changes in gene expression through the action of multi-protein nuclear complexes.

The nucleus, often referred to as the “brain” of the cell, is the site where genetic information is stored and where administrative decisions are made to direct growth, differentiation and metabolism in response to endogenous and environmental stimuli. Histones facilitate DNA packaging into structures called nucleosomes originally observed by electron microscopy as “beads on a string” [1,2]. Each nucleosome is composed of a core octamer containing two copies of each histone type H2A, H2B, H3, and H4 surrounded by 146 base pairs of DNA [3]. Core histones can be replaced by histone variants (H2A.Z, H2A.X, H3.1, H3.3) with specialized properties [4]. Amino-terminal histone tails are enriched in basic amino acids such as lysine (K) and arginine (R) that can be reversibly modified by acetylation, methylation, phosphorylation, sumoylation, ubiquitination, ADP ribosylation and other biochemical entities [4]. The deposition, removal, and recognition of specific post-translational modifications (PTMs) to histones is mediated by specialised enzymes known as writers, erasers, and readers, respectively. Specific histone PTMs can regulate the level of chromatin condensation or DNA accessibility, and the combination of these PTMs is known as the histone code.

The distinction between different chromatin areas was originally reported based on cytogenetic studies revealing visibly stained and highly condensed chromosomal regions, also known as heterochromatin, that contain transcriptionally inert genes or repetitive sequences [5]. On the contrary, chromatin areas appearing less-intensely stained and containing gene-rich regions were referred to as euchromatin [5]. A closer look at euchromatin showed compartmentalisation of local chromatin states with distinct transcriptional and epigenetic properties. In addition to actively transcribed genes and regulatory genomic regions enriched in histone acetylation, H3K4me3 and reduced nucleosome occupancy, euchromatin also contains genomic regions which are transcriptionally inert, enriched in DNA methylation, H3K27me3 and increased nucleosome density. The establishment and maintenance of heterochromatin and euchromatin at the molecular level at a sequence-specific context is regulated by the deposition of histone variants, histone modifications as well as DNA methylation, which play a major role in influencing chromatin structure, gene accessibility and nucleosome stability [6].

Major advances in next generation sequencing, leading to the assembly and annotation of plant genomes, and in epigenome profiling, have provided a huge amount of information essential for interpreting the genetic and epigenetic mechanisms of plant adaptation [7]. However, there is very limited information on how plant chromatin and multi-protein nuclear complexes are organized in the three-dimensional region of the nucleus. Dynamic sub-nuclear domains, where large numbers of proteins and nucleic acids assemble to perform specialized functions, have been observed in plants and metazoa [8,9]. The nucleolus acts as the site of ribosomal biogenesis and together with heterochromatic regions represent by far the most visibly distinctive sub-nuclear structures in plants. However, there are numerous uncharacterized plant nuclear bodies, whose function remains elusive [8, 9, 10].

Several studies have shown that the organization and positioning of nuclear structures such as the nucleolus and the chromocenters are not random [11,12]. The topology of fundamental and conserved nuclear processes such as transcription, splicing, DNA-damage repair and chromatin modifications is very important. Changes in the sub-nuclear localization of proteins and nucleic acid micro-domains have been correlated with certain genetic disorders and cancer [13,14]. In addition, the sub-nuclear positioning of genes has recently been shown to be involved in telomeric function and aging in humans [14]. However, it is still unclear whether disruption of nuclear organization is the cause or consequence of gene mis-regulation. A series of sophisticated techniques originally introduced in *Drosophil*a *melanogaster* and mammalian cells, have now been optimized and employed in plants to investigate nuclear compartments [15,16]. In particular, Chromatin Conformation Capture (3C), padlock Fluorescence In Situ Hybridization (FISH) and live three-dimensional (3D) imaging have opened new avenues to the field [15,16]. These techniques have already contributed to major scientific discoveries such as the existence of topologically associating chromatin domains (TADs) in large plant genomes and clustering of co-regulated genes [17, 18, 19].

The fundamental principles of chromatin organization and regulation of gene expression are conserved among kingdoms [20]. Primary studies in *Arabidopsis thaliana* and other plant species are leading the way to uncovering unique mechanisms shaping nuclear architecture and modulating transcriptional changes in gene expression in response to a constantly changing environment. Many excellent reviews discuss the impact of stress and nutrient availability on plant chromatin organization [7,21, 22, 23, 24, 25]. This review discusses recent progresses in our understanding of plant nuclear architecture and focuses on how two environmental stimuli, such as light and temperature, induce changes in chromatin organization correlating with regulation of gene expression. Major discoveries in the mechanisms controlling plant developmental transitions including photomorphogenesis and flowering, as well as acclimation that have been accomplished by employing revolutionary techniques such as Hi-C (3C coupled to next generation sequencing), high-resolution sub-cellular imaging combined with chromatin immunoprecipitation and conventional genetics will be discussed. Understanding how nuclear architecture responds to environmental fluctuations in light and temperature, and how plants cope with nutrient availability and stress will not only provide the tools and knowledge to increase crop productivity, but will also allow biologists to understand key biological processes in a tissue-specific context at the nano-scale level [16,26,27].

## Novel discoveries light up plant nuclear architecture

The eukaryotic nucleus allows communication and controlled transport between the cytosol and the nucleoplasm through the nuclear pore complex (NPC), which is embedded in the nuclear envelope (NE). The interior of the plant NE is supported by a matrix of proteins with analogous properties to the metazoan lamins in maintaining nuclear structure and influencing chromatin organization and gene expression [28]. Seminal studies in yeast, flies and mammals have shown that lamins help with the establishment of sub-nuclear, chromosomal domains characterized by distinct nucleosomal arrangement, epigenetic composition and transcriptional — mostly repressive — properties and referred to as lamin-associated domains (LADs) [14,29,30]. Lamins are thought to act as matrix-tethering LAD regions to the nuclear periphery facilitated by transcription factors and histone deacetylases [14]. The mammalian *CYSTIC FIBROSIS TRANSMEMBRANE CONDUCTANCE REGULATOR* (*CFTR*) locus is repressed by relocation to the nuclear periphery and anchoring to lamins by directly associating with the CCCTC-binding factor (CTC) and a HDAC [14]. On the contrary, specific components of the NPC tend to attract actively transcribed loci and facilitate the nuclear export of nascent mRNAs [14]. Yeast provides a great example where gene repositioning to the nuclear periphery directly correlates with transcriptional activation. Yeast genes that contain a consensus promoter sequence or “DNA code” are recognized and recruited to the nuclear periphery by specific transcription factors that interact with nuclear pore complex components (NUP) [14].

**Figure 1:**
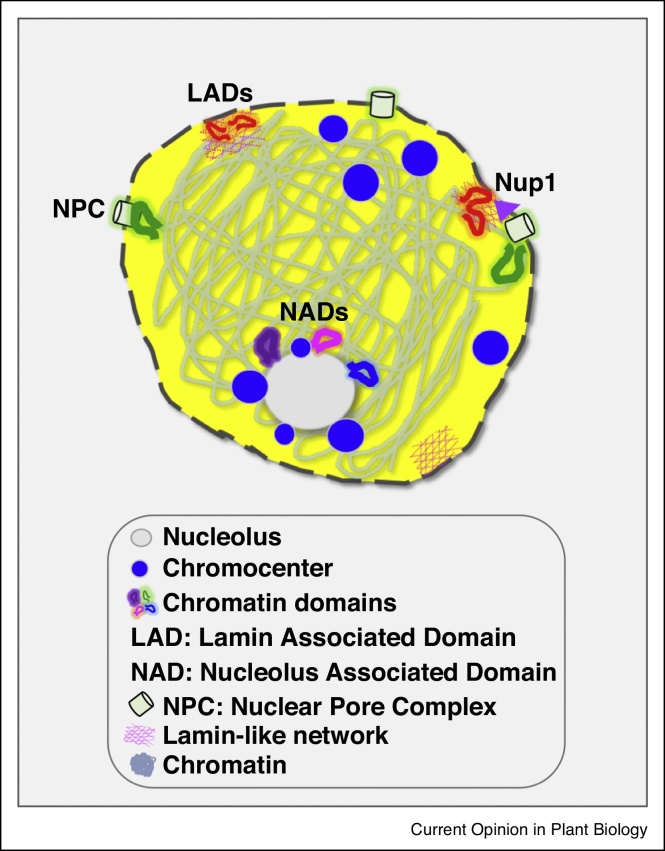
Illustration of the main features defining plant nuclear architecture. Nucleo-cytoplasmic communication and trafficking is achieved through the action of the nuclear pore complex (NPC). A lamin-like matrix provides support and docking sites for lamin-associated chromatin domains (LADs) [29,30]. LADs and nucleolar-associated domains (NADs) are enriched in chromocenters, repetitive DNA sequences, transposable elements and silenced loci [33]. Recent discoveries showed that transcriptionally active and highly expressed genes associate with the nuclear periphery [35^••^] and NPC components [31]. The existence of topologically associating domains (TADs) have only been observed in selected crop species [20,57^••^,^58^].

Recent studies developing a modified chromatin immunoprecipitation (ChIP) protocol using an epitope-tagged plant NPC component (NUP1-GFP) known to interact with the lamin-like matrix revealed that the nuclear periphery of Arabidopsis is enriched in heterochromatin as well as repressed coding genes [31]. Correlation of NUP1-associated sequences with global chromatin interaction maps obtained by Hi-C, FISH as well as histone and DNA methylation patterns indicate that specific areas of the Arabidopsis nuclear periphery are primarily associated with transcriptionally repressed regions [31]. These data agree with initial observations where plant chromocenters (centromeres and pericentromeric regions containing condensed chromatin) have been localized in the nuclear periphery (Figure 1) [32].

A separate study based on the sequencing of DNA associated with isolated nucleoli, sorted by fluorescence-based flow cytometry, identified nucleolus-associated domains (NADs) [33]. In addition to rRNA, NADs show enrichment in telomeres, heterochromatin, repressed genes and transposable elements (Figure 1). Interestingly, alterations in the nucleolar structure lead to shorter telomeric repeats with direct implications in plant growth, development and fertility [33].

Quantitative 3D imaging of Arabidopsis nuclei has recently revealed that a cluster of proteins linking the NE with the cytoskeleton (LINC complex) are important for anchoring plant chromocenters to the nuclear periphery [34^•^]. In particular, LINC mutants exhibit not only altered nuclear morphology and chromocenter topology, but also abnormal activation of normally transcriptionally repressed heterochromatic regions. This study indicates that the protein components of the plant NE are important for regulating chromatin organization and activity (Figure 1) [34^•^]. It is worth mentioning that several light-induced genes such as *CHLOROPHYLL A/B-BINDING* (*CAB*), *RUBISCO SMALL SUBUNIT* (*RBCS*), *PLASTOCYANIN* (*PC*) and *GENOMES UNCOUPLED 5* (*GUN5*) have been observed to re-position to the nuclear periphery in response to light [35^••^]. Tethering experiments of reporter genes to a plant nucleoporin showed an increase in gene expression [36], whereas NUP1-associated sequences were enriched in repressed genes [31].

These studies indicate that the plant NE potentially holds a dual role in regulating gene expression depending on the chromatin context and precise topology on the nuclear periphery (NPC versus lamin-like protein association) [31,35^••^,^36^]. Although yeast and mammalian systems have provided key examples demonstrating a direct association between gene repositioning to the nuclear periphery with gene activation or repression, there is very limited information correlating nuclear positioning and gene expression in plants [14,35^••^]. Future molecular, genetic and imaging studies are required to test the causality between locus-specific transcriptional regulation and anchoring at the NE, perhaps by identifying the plant nuclear components required for tethering.

## Light triggers dynamic changes in the plant nucleus

The spectrum of sunlight ranges from invisible ultra-violet irradiation to visible wavelengths of light. Plants absorb photons through the action of specialized photoreceptors (UV-B: UV-RESISTANCE LOCUS 8; blue: cryptochromes, phototropins, zeitlupes; red/far-red: phytochromes) that localize in most plant organs, whereas animal photoreceptors localize in specialized visual organs [37]. Light quality, quantity, direction and duration provide invaluable information that is essential for triggering major developmental and architectural transitions in plants. In addition to modulating hormone gradients, light drives major transcriptional re-programming through the coordinated action and nuclear clustering of key, shared signaling components and transcriptional regulators [9,10,38,39].

### Histone modifications underlying light regulation of gene expression

**Figure 2:**
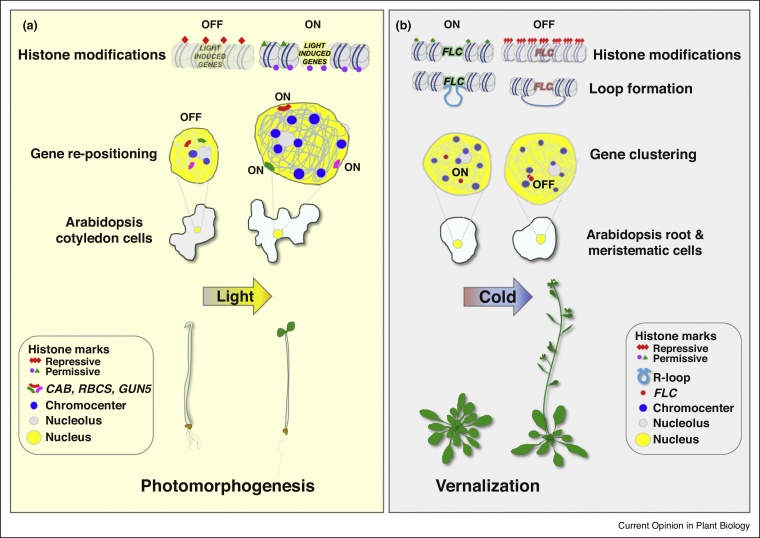
Overview of two examples where environmental stimuli trigger short or long-distance changes in chromatin organization. **(a)** Light mediates changes in histone modifications, gene relocation to the nuclear periphery and an increase in nuclear size and DNA content through the action of photoreceptors and light signaling components [35^••^,^42^,44^••^,^64^,^65^]. Light-induced changes in nuclear architecture lead to the transcriptional activation of light-responsive genes essential for photomorphogenesis (*CAB*, *RBCS*, *GUN5*) [35^••^,44^••^]. **(b)** Prolonged cold temperatures are required for flowering initiation by silencing of the negative regulator of flowering *FLC*. Prolonged cold triggers *FLC* gene clustering and disruption of gene loop formation followed by epigenetic silencing of *FLC* through the deposition of repressive histone marks [53,54^••^].

A range of histone modifications and chromatin remodelling enzymes have been shown to play important roles in mediating light-regulated gene expression in Arabidopsis [7,10,40]. Histone acetylation and methylation have been associated with the induction and repression of light-responsive loci leading to changes in hypocotyl growth through the action of histone de-acetylases (HDA19, HDA15), acetyl-transferases (GCN5), methyl-transferases (SDG8) as well as de-methylases (JMJ20, JMJ22) and chromatin remodellers (PICKLE) [10,40,41]. More recent studies have revealed the first report in plants assigning a physiological and molecular function to H2B mono-ubiquitination (H2Bub) in regulating photomorphogenesis, which is the transition from dark to light-induced plant development [42]. The levels of H2Bub deposition positively correlate with the induction of major light signal integrating components, whereas photoreceptors and photosynthetic enzymes were unaffected (Figure 2a) [42]. The significance of a possible cross-talk between H2Bub and other histone modifications, such as methylation, which has previously been reported in yeast, remains to be investigated [43]. Furthermore, the role of light quality, quantity, photoperiod and potentially temperature oscillations in regulating this process remain unclear. Does H2Bub affect the accessibility of light-regulated genes to specific transcription factors? Which light wavelengths and photoreceptor families regulate H2Bub deposition? Does this modification act as a rheostat adjusting gene expression in response to the environment? Does H2Bub precede or is it a consequence of light-regulated changes in chromatin organization?

### Light induces changes in genome topology

In addition to mediating changes in histone mark deposition, light can also have a direct role in triggering the movement of gene loci in 3D. An innovative padlock FISH protocol was applied in Arabidopsis nuclei and revealed that light triggered preferential re-positioning of light-inducible photosynthetic genes (*CAB*, *GUN5*, *PC*) from the nucleoplasm to the nuclear periphery (Figure 2a) [35^••^]. Gene relocation to the nuclear periphery was correlated to an increase in transcript abundance verified by quantitative gene expression analysis. The red-light receptor phyB, key light signaling components (COP1: CONSTITUTIVE PHOTOMORPHOGENIC 1, DET1: DE-ETIOLATED 1) and, to a lesser extent, transcription factors play a role in mediating the relocation of the afore-mentioned loci in response to light [35^••^].

As previously discussed, the NPC has been associated with active transcription by facilitating newly-synthesized mRNA export in metazoa and yeast, whereas the nuclear periphery in general has been associated both with active gene expression or silencing [14,31,35^••^,^36^]. Light-induced gene relocation therefore provides the first piece of evidence linking environmentally-induced activation of gene expression with the nuclear periphery in plant cells [35^••^]. Whether these light-regulated loci directly associate with subunits of the NPC remains to be determined. More importantly, do these light-regulated loci contain a common “DNA code” in their promoters that could potentially be recognised by specific transcription factors that facilitate their recruitment to the NPC in a similar manner to several yeast genes [14]? It will be interesting to examine if non-photosynthetically-related light-induced genes also exhibit re-positioning to the nuclear periphery, and if light-repressed loci follow the opposite pattern.

### Nuclear reorganization in response to blue and red light

To investigate how light mediates large-scale changes in nuclear morphology and genome-wide chromatin organization, and how these changes correlate with the regulation of gene expression and plant development, a combination of cytogenetic and next generation sequencing approaches have been employed in Arabidopsis [44^••^,45^•^]. The key light signaling components COP1 and DET1 were shown to have major functions in mediating heterochromatin relaxation prior to a seedling’s initial exposure to light [44^••^]. Upon photomorphogenesis, light triggers the gradual condensation of heterochromatin leading to a significant increase in nuclear expansion, and the appearance of chromocenters that are clearly visible in cotyledon cells of five-day old seedlings (Figure 2a) [44^••^]. This response is primarily induced by blue and, to a lesser extent, red light through the action of cryptochromes and phyB, respectively. No correlation was observed between heterochromatin condensation and DNA methylation, whereas an increase in RNA Pol II activity was measured in response to light [44^••^]. These data clearly indicate that germination is accompanied by chromatin decondensation, followed by light-regulated chromatin re-condensation during cotyledon differentiation and leaf maturation [46]. A reciprocal phenomenon is observed when mature plants are grown under low-intensity light and prior to flowering, where cry2 triggers heterochromatin decondensation in leaf cells [46]. To understand how photoreceptors trigger diverse chromatin changes during germination, de-etiolation and flowering would require more detailed analysis at a tissue or even cell type-specific level. Do photoreceptors mediate light-regulated changes in chromatin architecture by associating with specific protein complexes and chromatin remodeling machinery at different developmental stages, tissues and cell-types?

A recent report examining the nuclear size and heterochromatin index in different Arabidopsis accessions has assigned a novel role to the red light receptor and thermosensor, phyB, in negatively regulating nuclear expansion and heterochromatin condensation [45^•^]. High temperature is also known to trigger chromatin decondensation in plant nuclei [21]. Future studies focusing on how phyB and other photoreceptors control changes in nuclear architecture and how these changes correlate with the transcriptional regulation of gene expression in response to light and temperature could provide a better understanding of signal integration and output in repose to a constantly changing environment.

## Temperature fluctuations impact local nuclear organization

Temperature is an important environmental parameter that regulates heterochromatin compaction within the plant nucleus [12]. More specifically, heat stress has been shown to lead to a reduction in nucleosome density, which is primarily regulated by the chromocenter-localized protein HEAT-INTOLERANT 4 (HIT4) [12,47]. The intimate relationship between light and temperature was formally solidified through the discoveries that Arabidopsis phyB and *Marchantia polymorpha* phototropin can act as thermosensors due to thermo-regulation of their photocycle kinetics [38]. Moreover, an increasing number of seminal research articles demonstrate how light and temperature pathways integrate through shared signaling components and transcriptional hubs that modulate gene expression [38,48]. Whether changes in ambient temperature can modulate the photochemistry, signaling activity, post-translational modifications and protein interactome of all plant photoreceptors remains to be investigated. In particular, photoreceptors such as phytochromes and cryptochromes that are involved in light-mediated changes in nuclear architecture and gene expression could also play a role in modulating temperature-mediated chromatin decondensation. The role of photoreceptors and light signaling components in regulating the expression of chromatin remodelling enzymes in response to light [49] or temperature is limited, but currently flourishing and could provide molecular insights in understanding the mechanisms underlying chromatin organization in response to the environment.

### Chromatin remodelling in response to elevated temperature

Thermomorphogenesis refers to a set of morphological and developmental responses (elongated hypocotyls, stems and petioles; early flowering) resulting from an increase in ambient temperature. Plant thermomorphogenic phenotypes resemble the shade avoidance response, both of which are partly mediated by phyB-regulated changes in gene expression [48]. As previously mentioned, elevated temperature leads to chromatin decondensation [12]. The role of chromatin remodelling in regulating thermomorphogenesis through the “eviction” of the histone variant H2A.Z was a revolutionary finding in the plant field [48]. Heat shock factors (HSF1) have recently been shown to directly bind to the transcriptional start site of genes induced by warm temperatures (26–28 °C) and act as a prerequisite for the removal of H2A.Z leading to transcriptional activation [50].

New evidence is emerging in examining the function of histone modifications in inducing thermo-regulated gene expression. The chromatin remodelling factor PICKLE (PKL), previously shown to act as a negative regulator of photomorphogenesis, has an active role in promoting hypocotyl elongation in response to high temperature. More specifically, PKL modulates the methylation status of H3K27 and activates the expression of auxin-responsive and growth-promoting genes essential for hypocotyl growth specifically at 28 °C. PKL bridges not only light and temperature signaling, but also circadian rhythms, as the CIRCADIAN CLOCK-ASSOCIATED 1 (CCA1) component is reported to associate with the *PKL* promoter and induce its transcript accumulation [51^•^]. Future research investigating the role of different histone modifications and transcriptional regulators in controlling genome-wide chromatin patterns in response to light, clock, hormone and temperature changes is needed for understanding the molecular mechanisms of signal integration at the chromatin level. Resolving the temporal and spatial dynamics of multi-stimulus induced chromatin re-organization and transcriptional regulation of gene expression could be challenging, but very informative. More specifically, what determines the rapidity of light or temperature-induced changes in gene expression? Do photoreceptors, transcriptional regulators and chromatin remodelling enzymes cluster as large multi-protein complexes in the vicinity of coregulated genes [10]? Are there intrinsically disordered scaffold proteins that facilitate signal integration through protein interactions near specific chromatin domains? Does the plant nucleus exhibit liquid-liquid phase separation to control nuclear organization in response to environmental stimuli [52]?

### Cold-induced changes in chromatin conformation

Vernalization, also known as the requirement of a prolonged cold winter period prior to inducing flowering initiation, is by far one of the best characterized epigenetic silencing pathways in response to environmental stimuli. Winter temperatures lead to silencing of *FLOWERING LOCUS C* (*FLC*) through the action of long non-coding RNAs, followed by the progressive deposition of chromatin repressive marks by the Polycomb Repressive Complex 2 (PRC2) (Figure 2b) [53]. Short-distance chromatin loops, as well as irreversible, cold-induced long-distance interactions of the Arabidopsis *FLC* loci have been observed using 3C and live imaging, respectively [53,54^••^]. In the latter case, *FLC* clustering showed a direct correlation with cold-induced epigenetic silencing through deposition of H3K27 trimethylation (Figure 2b) [53,54^••^]. However, the molecular and cellular mechanisms regulating such long-distance chromatin clustering in response to vernalization remains to be elucidated. Are there specific histone modifying enzymes that facilitate clustering to predefined sub-nuclear compartments?

Experiments performed in field conditions have recently uncovered a dual mechanism for determining the right time for flowering initiation in response to temperature changes. Sensing the absence of elevated temperature oscillations is as important as the perception of prolonged cold, for regulating *FLC* silencing and ultimately inducing flowering initiation [55]. Therefore, future studies focused on determining signal integration at the chromatin level are essential to understand the molecular basis of plant responses to a constantly changing natural environment.

### Genome-wide chromatin changes in response to cold

Pioneering work using genome-wide Hi-C technologies in Arabidopsis have provided quantitative information on chromosomal interactions and genome organization in high resolution [56]. To address how temperature fluctuations impact global chromatin structure in 3D, very elegant Hi-C experiments were performed on rice that led to two major discoveries: (a) unlike Arabidopsis, rice chromatin exhibits organization in TADs similar to animal genomes, and (b) exposure to low temperature induces weakening specifically of long-range chromatin interactions leading to chromatin decondensation [57^••^]. Similar studies on chromatin structure, histone and DNA modifications in crop species, such as maize, sorghum and tomato, have also revealed that specific plant genomes are organized into euchromatin/heterochromatin regions resembling a feature conserved in metazoa [58]. Furthermore, plant genomes exhibit chromatin loop formation near the TAD borders that correlate with active gene expression, unlike mammalian loops [58]. Overall, plants provide a great system to study species-specific features in hierarchical genome organization in 3D. The use and application of Hi-C technologies in plants is currently flourishing and has already led to unexpected and exciting discoveries that will undoubtedly contribute to our understanding of how plants respond to their environment at the chromatin level.

## Conclusions

Plants are exposed to a variety of environmental stimuli that shape plant architecture at every level. Light and temperature are two major parameters determining the developmental fate and growth pattern of a plant by regulating gene expression. Recent revolutionary studies have clearly shown how changes in light and temperature can induce localized changes in chromatin composition and 3D structure, as well as regulating long-distance genome topology and global nuclear architecture. The molecular mechanism linking genome topology to transcriptional activation or repression is currently being uncovered in yeast, Drosophila and mammals by employing novel high throughput screening [14,59,60]. Although there are significant studies correlating gene re-positioning with transcriptional activity in plants, we are still far from understanding the underlying mechanism and the key players driving such local and genome-wide changes in nuclear architecture. Do plants contain specialized protein hubs that orchestrate genome organization in 3D? Are there predefined sub-nuclear domains that act as anchoring docks for proteins and coregulated loci? Are there nuclear hubs where genes become silenced or actively transcribed in response to dynamic changes in light, temperature and nutrient availability? Is there a tissue and cell specific nuclear organization signature and how/when is it acquired? The discovery and application of cutting-edge technologies in super-resolution imaging, genome editing and chromatin conformation capture, coupled to high-sensitivity next generation sequencing and mass spectrometry, are leading the field of plant nuclear architecture to a period of “enlightenment” [16,18,19,26,61, 62, 63].

## References and recommended reading

Papers of particular interest, published within the period of review, have been highlighted as

• of special interest
•• of outstanding interest
